# Is robotic surgery beneficial for the treatment of endometrial cancer? A comparison with conventional laparoscopic surgery

**DOI:** 10.7150/jca.88187

**Published:** 2024-01-01

**Authors:** Joo Hee Yoon, Chae Young Yun, Sujin Choi, Dong Choon Park, Sang Il Kim

**Affiliations:** Department of Obstetrics and Gynecology, St. Vincent's Hospital, College of Medicine, The Catholic University of Korea, Seoul, Republic of Korea.

**Keywords:** endometrial cancer, uterine cancer, minimally invasive surgery, robotic surgery, conventional laparoscopic surgery

## Abstract

**Objective:** The objective of this study was to compare the oncologic outcomes between those who underwent robotic surgery or conventional laparoscopic surgery for endometrial cancer.

**Method:** We performed a retrospective review of patients from a single institution who underwent either laparoscopic or robotic surgery for the treatment of endometrial cancer between January 2010 and December 2020. Tumor characteristics, recurrence rate, disease-free survival, and overall survival were compared according to surgical approach.

**Results:** Among the 268 patients included in this study, 95 underwent robotic surgery (35.4%) and 173 underwent laparoscopic surgery (64.6%). The median follow-up durations were 51 and 59 months for the robotic surgery and laparoscopic surgery groups, respectively (*p* = 0.085). The recurrence rate did not differ significantly between the two groups. (*p* = 0.371). Disease-free survival (*p* = 0.721) and overall survival (*p* = 0.453) were similar between the two groups. In both univariate and multivariate analyses, the type of surgery was not related to disease-free survival. The median total cost per admission was significantly higher for RS than for LS (12,123 vs. 6,884 USD, *p* < 0.0001).

**Conclusion:** With consistently greater costs and similar survival outcomes, robotic systems have few advantages compared with laparoscopy.

## Introduction

Endometrial cancer (EC) is the most common gynecologic cancer, with the estimated number of cases in Korea being over 3,000 by 2023 1. Treatment guidelines for newly diagnosed EC are well-established 2, 3. Surgery is often the primary treatment for EC, except for patients with distant metastases. In contrast to cervical cancer, minimally invasive surgery (MIS) is an acceptable treatment for EC 4. The safety of MIS for EC has been supported in many randomized clinical trials (RCTs) 5-8, which have reported similar oncologic outcomes. a lower rate of complications, and a shorter hospital stay. Consequently, the application of MIS has gradually increased 9.

Two surgical approaches are classified as MIS: conventional laparoscopic surgery (LS) and robotic surgery (RS). In 2005, the Food and Drug Administration approved RS for gynecological indications. Since then, the proportion of EC treated via RS has been increasing, thereby replacing LS 10. As a result, approximately 80% of patients underwent RS for EC in the United States to date 11.

The advantages of RS over LS include improved three-dimensional visualization, more ergonomic surgeon position, and articulated wrist-like instruments, thereby increasing surgical precision and dexterity 12. Numerous studies have compared RS and LS, and most have focused on their safety and complications 13-16. Only a few studies have compared the oncological outcomes of RS and LS. Thus, the advantages of RS over LS in EC treatment are yet to be fully determined. Considering the high cost of purchasing and maintaining these robotic systems, it is important to demonstrate the benefits of RS compared with LS 17.

We decided to evaluate the data from our institution to compare survival outcomes in a cohort of women undergoing RS and LS for EC.

## Materials and Methods

This retrospective cohort study was approved by the Institutional Review Board of the Catholic University of Korea (VC23RASI0258). The requirement for informed consent was waived because of the retrospective nature of the study.

Our institution's cancer registry was reviewed to identify patients diagnosed with endometrioid and non-endometrioid EC between January 2010 and December 2020 at St. Vincent Hospital. Medical records, pathology reports, imaging studies, information about clinicopathological characteristics (age, histologic type, grade, stage, tumor size, and other risk factors), and adjuvant treatments were collected and reviewed for all patients. Gynecologic oncologists performed all surgeries. Only the patients who underwent primary surgery via RS or LS were eligible. All patients underwent at least a complete hysterectomy, while both pelvic and para-aortic lymphadenectomies were recommended. A lymphadenectomy was recommended based on risk factors and the preoperative and intraoperative evaluations. All patients who underwent RS used the da Vinci robotic system (Intuitive Surgical, Inc. 1266 Kifer Road, Building 101 Sunnyvale, CA). Postoperatively, adjuvant therapy, either radiotherapy or chemotherapy, was selectively administered according to the disease stage and risk factors. The exclusion criteria included the following: (1) patients who underwent open surgery; (2) patients primarily treated with medication or with radiation alone; (3) distant metastasis (stage IVB); and (4) patients with insufficient clinical and/or pathology data.

Patients who met the inclusion criteria were divided into two groups: those who underwent RS and those who underwent LS.

Disease-free survival (DFS) was calculated from the date of surgery to that of recurrence based on the pathological or radiologic confirmation or the date of the last follow-up. Overall survival (OS) was calculated from the date of surgery to that of cancer-related death or the last follow-up.

Differences in the clinicopathological characteristics between the two groups were evaluated.

The chi-squared and Fisher's exact tests were performed to compare categorical variables. Continuous variables were compared using Student's t-test or the Mann-Whitney test. We performed the Kaplan-Meier method with log-rank tests to compare DFS and OS between the two groups. Univariate logistic regression analysis was performed using the Cox proportional hazards model to analyze the effects of the prognostic factors. Multivariate logistic regression analysis was subsequently used to estimate the odds ratio (OR) for each covariate.

All statistical analyses were performed using the SPSS statistical software (version 21.0; SPSS Inc., Chicago, IL, USA). Statistical significance was defined as a *p*-value of <0.05.

## Results

The data of 427 patients were reviewed; a total of 268 were identified as having endometrioid or non-endometrioid EC and were included in the final analysis. Of these patients, 95 (35.4%) underwent RS, and 173 (64.6%) underwent LS. The clinicopathological characteristics of the patients are summarized in Table 1. The mean age and body mass index of the patients were comparable between the groups. We used the guidelines of the 2009 International Federation of Gynecology and Obstetrics (FIGO) to define cancer stages. Neither group showed a significant difference in the FIGO stage, histological subtype, or grade. Patients who underwent LS had larger tumors (mean size, 2.69 vs. 2.13 cm, *p* = 0.010) and a higher depth of myometrial invasion (DMI) rate (24.3% vs. 11.6%, *p* = 0.016). The two groups were comparable in terms of lymphovascular space invasion rate and lymphadenectomy status. The proportions of patients receiving adjuvant therapy and treatment regimens did not differ significantly between the groups.

Table 2 shows the median hospital charges for surgery. The median total cost per admission was significantly higher for RS than for LS (12,123 vs. 6,884 USD, *p* < 0.0001). As RS is not covered by the Korean National Health Insurance Service (NHIS), patients who received RS incurred significantly higher costs than those who received LS (9,155 vs. 2,567 USD, *p* < 0.0001).

The median follow-up durations were 51 and 59 months for the RS and LS groups, respectively (*p* = 0.085). Overall, 24 patients (9.0%) experienced disease recurrence. Recurrence occurred in 6 (6.3%) of the 95 RS cases and 18 (10.4%) of the 173 LS cases. The recurrence rate was higher in the LS group but did not differ significantly between the two groups. (*p* = 0.371). There were eight (3.0 %) cancer-related deaths in the overall population: 1 (1.1 %) in the RS group and seven (4.0 %) in the LS group (*p* = 0.267).

**Figure 1:**
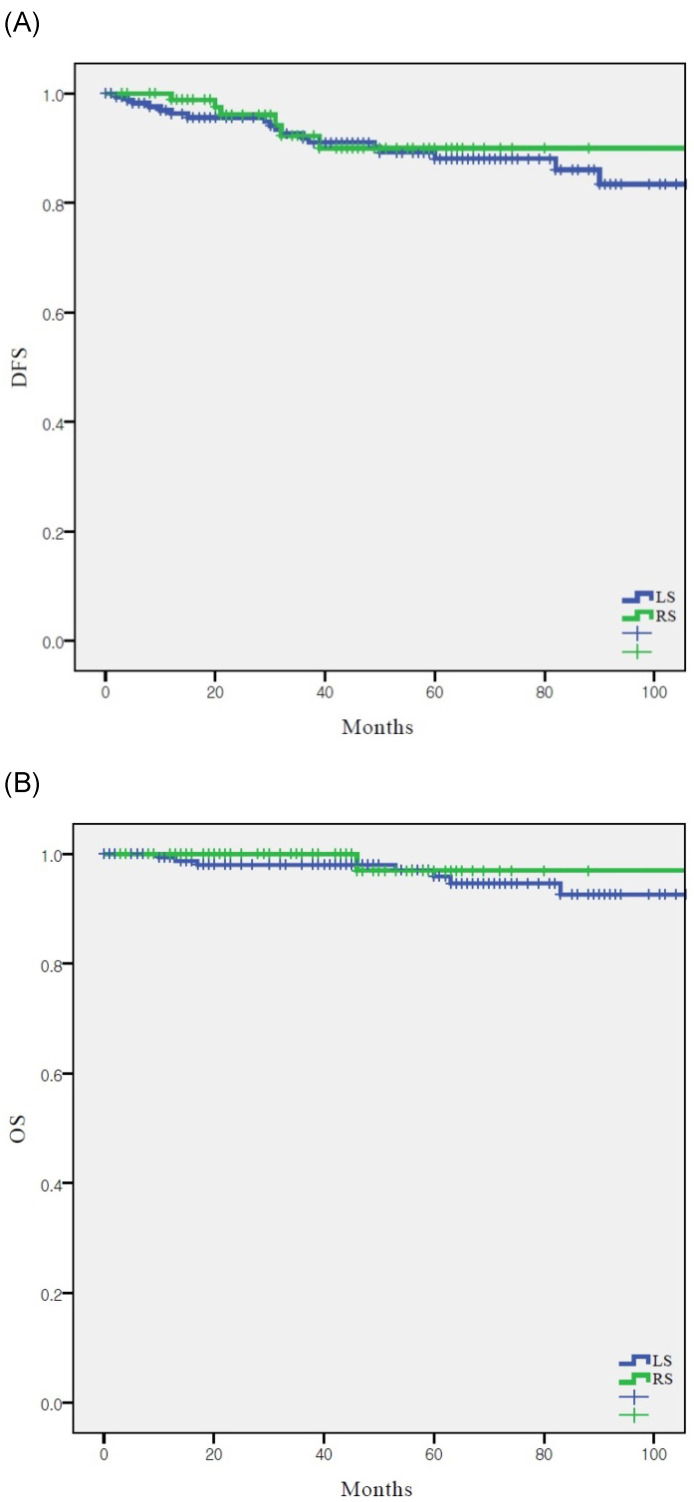
Survival outcomes according to surgical approach (n = 268) (A) disease-free survival in entire cohort, (B) overall survival in entire cohort

In the overall population, DFS (*p* = 0.721) and OS (*p* = 0.453) were similar between the two groups (Fig. 1). The 5-year DFS and OS rates were 90.0% and 96.9% in the RS group and 88.1% and 95.9% in the LS group, respectively.

Univariate and multivariate analyses of clinicopathological variables are shown in Table 3. In univariate analysis, histologic grade, DMI, and adjuvant radiotherapy were significantly associated with DFS. However, the type of surgery was not related to DFS. In multivariate logistic regression analysis, grade, and DMI were identified as the risk factors for DFS.

## Discussion

In this study, we compared the oncologic outcomes of RS and LS. Among women with EC, RS and LS showed similar recurrence and survival outcomes. These results are consistent with those of previous studies 18-21.

The advantages of MIS in treatment of EC have been well-established in multiple RCTs 5-8. MIS showed a lower complication rate in these studies without compromising survival outcomes. Based on the positive results of these studies, the standard of care for EC has shifted from open surgery to MIS. RS systems were developed later than LS; therefore, these studies did not include RS. Although numerous studies have compared RS and LS for the treatment of EC, the main focus of these studies has been safety and complications 13-16. Moreover, RS is reportedly equivalent to LS in terms of intra- and postoperative complications and length of hospital stay. Few studies have compared survival outcomes between RS and LS for EC, suggesting similar survival outcomes 18-21. Most of the earlier studies that compared survival outcomes between RS and LS for EC reported 3-year or 5-year DFS and OS. Cardenas-Goicoechea et al. reported no significant differences in survival outcomes between RS and LS (3-year DFS was 88.4% and 83.3% and 3-year OS was 93.6% and 93.3%, respectively) 18. Similarly, Corrado et al. reported 3-year DFS rates of 91.5% and 88.4% and 3-year OS rates of 91.5% and 91.7% for RS and LS, respectively 19. Moreover, Eoh et al. reported comparable survival outcomes between the RS and LS groups (5-year DFS, 93.1 vs. 92.3 %, and 5-year OS, 94.8 vs. 91.9 %) 20. However, Argenta et al. recently reported inferior 10-year survival outcomes with RS compared with those with LS for the treatment of stage I EC 22. In this study, more than half of the recurrences occurred at >24 months postoperatively (55% in the RS group and 68% in the LS group). Moreover, 24% of the patients in the RS group and 28% in the LS group experienced recurrence after at least 5 years. Thus, previously reported 3-year or 5-year outcome data may underestimate long-term survival outcomes.

The advantages of RS are well known. The ergonomic console has a wide range of motion and rotation, increasing surgical precision and dexterity 23. However, these advantages did not lead to superior outcomes over LS. Moreover, Argenta et al. reported poorer long-term patient outcomes with RS than with LS in the treatment of stage I EC, which contradicts the results of a previous study 22.

In our cohort, we observed no differences in clinicopathological characteristics, except tumor size and DMI, between the two groups. Tumor size was significantly larger in the LS group, and the rate of DMI was higher in the LS group. Recurrence rates in the RS and LS groups were 6.3% and 10.4%, respectively. Although the recurrence rate was not significantly different between the two groups, it was slightly higher in the LS group. Because tumor size and DMI are well-known risk factors in patients with EC, the higher recurrence rate in the LS group can be explained.

One of the main disadvantages of RS compared with LS is its higher cost 24-26. The acquisition fee for robotic systems is higher than that for laparoscopy. In addition, robotic instruments have a limited number of lives, and the expense of instrument replacement is high. Additionally, RS is not covered by the NHIS in South Korea, whereas LS is fully covered. Thus, in concordance with the results of our study, RS was associated with a considerably higher total cost per admission and price paid by the patient.

Our study has several limitations. First, owing to the retrospective study design, there may have been inevitable issues such as selection bias. Second, the relatively small sample size and short observation period may have been insufficient for appropriate comparison of the oncologic outcomes between the two groups. The median observation times in our study were approximately 51 and 59 months for the RS and LS groups, respectively. Because the robotic system was adopted later, the observation time was shorter in the RS group. Third, although all surgeries were performed by gynecologic oncology specialists, surgical competence and proficiency among the surgeons were not considered. Lastly, perioperative complications and morbidity rates were not evaluated.

In conclusion, RS showed comparable survival outcomes to LS in women with EC. However, the high cost of purchasing and maintaining robotic systems must also be considered. With consistently greater costs and similar survival outcomes, robotic systems have few advantages compared with laparoscopy. Further large-scale RCTs and clinical studies are required to provide relevant data.

## Data Availability

The data that support the findings of this study are available on request from the corresponding author.

## Figures and Tables

**Table 1:** Clinico-pathological characteristics of patients according to surgical approach (n = 268)

	Total (n = 268, %)	RS (n = 95, %)	LS (n = 173, %)	*p-*value
Age (years), mean ± SD	53.87 ± 10.52	51.66 ± 9.74	55.08 ± 10.76	0.423
BMI (kg/m^2^), mean ± SD	25.52 ± 4.42	25.58 ± 4.27	25.49 ± 4.51	0.938
FIGO stage				0.118
I	236 (88.1)	88 (92.6)	148 (85.6)	
II	10 (3.7)	2 (2.1)	8 (4.6)	
III	19 (7.1)	3 (3.2)	16 (9.2)	
IV	3 (1.1)	2 (2.1)	1 (0.6)	
Grade				0.086
1	148 (55.2)	61 (64.2)	87 (50.3)	
2	84 (31.3)	23 (24.2)	61 (35.3)	
3	36 (13.4)	11 (11.6)	25 (14.4)	
Histology				0.776
Endometrioid	255 (95.1)	90 (94.7)	165 (95.4)	
Non-endometrioid^*^	13 (4.9)	5 (5.3)	8 (4.6)	
Tumor size (cm), Mean ± SD	2.50 ± 1.69	2.13 ± 1.47	2.69 ± 1.77	0.010^+^
DMI ≥ 50%	53 (19.8)	11 (11.6)	42 (24.3)	0.016^+^
LVSI positive	28 (10.4)	6 (6.3)	22 (12.7)	0.142
Lymphadenectomy	223 (83.2)	75 (78.9)	148 (85.5)	0.175
Adjuvant treatment				0.188
None	168 (62.7)	66 (69.5)	102 (59.0)	
Radiotherapy	82 (30.6)	25 (26.3)	57 (32.9)	
Chemotherapy	18 (6.7)	4 (4.2)	14 (8.1)	

^*^Serous, clear cell, carcinosarcoma, ^+^*p-*value < 0.05 RS, robotic surgery; LS, conventional laparoscopic surgery; SD, standard deviation; BMI, body mass index; FIGO, International Federation of Gynecology and Obstetrics; DMI, depth of myometrial invasion; LVSI, lymphovascular space invasion

**Table 2:** Median charges per admission according to surgical approach (n = 268)

	RS (n = 95, %)	LS (n = 173, %)	*P* value
^*^Total cost, median	12123	6884	< 0.0001^+^
Range	7286 - 17614	2166 - 20786	
^*^Paid by patients, median	9155	2567	< 0.0001^+^
Range	6436 - 15189	315 - 13318	
^*^Paid by NHIS, median	2881	4184	< 0.0001^+^
Range	0 - 6289	0 - 15571	

^*^USD, ^+^*p-*value < 0.05 RS, robotic surgery; LS, conventional laparoscopic surgery; NHIS, National Health Insurance Service

**Table 3:** Univariate and multivariate analysis of prognostic factors for disease-free survival (n = 268)

Characteristics	Univariate analysis			Multivariate analysis		
	OR	95% CI	*p value*	OR	95% CI	*p value*
Age	1.033	0.989 - 1.079	0.142			
BMI	0.950	0.849 - 1.063	0.374			
Surgical approach						
LS	1 (Ref)	-	-			
RS	0.792	0.257 - 2.443	0.685			
FIGO stage						
I/II	1 (Ref)	-	-			
III/IV	1.711	0.508 - 5.766	0.386			
Histologic type						
Endometrioid	1 (Ref)	-	-			
Non-endometrioid^*^	8.732	0.735 - 103.76	0.086			
Grade						
1	1 (Ref)			1 (Ref)		
2	4.432	1.452 - 13.53	0.009^+^	3.763	1.312 - 10.80	0.014^+^
3	8.006	1.779 - 36.02	0.007^+^	4.305	1.148 - 16.14	0.030^+^
DMI						
< 50%	1 (Ref)	-	-	1 (Ref)	-	-
≥ 50%	2.773	1.105 - 10.49	0.009^+^	3.944	1.323 - 11.76	0.014^+^
Tumor size	1.483	0.773 - 1.989	0.113			
LVSI						
Negative	1 (Ref)	-	-			
Positive	4.273	0.761 - 23.00	0.099			
Adjuvant treatment						
None	1 (Ref)	-	-	1 (Ref)	-	-
Radiotherapy	0.201	0.051 - 0.800	0.023^+^	0.295	0.086 - 1.012	0.052
Chemotherapy	0.197	0.016 - 2.364	0.200	0.691	0.158 - 3.027	0.624

^*^Serous, clear cell, carcinosarcoma, ^+^*p-*value < 0.05 OR, odds ratio; CI, confidence interval; Ref, reference; LS, conventional laparoscopic surgery; RS, robotic surgery; DMI, depth of myometrial invasion; LVSI, lymphovascular space invasion
